# Efficacy of noninvasive ventilation in patients with COVID-19

**DOI:** 10.1186/s13054-022-04241-4

**Published:** 2022-11-29

**Authors:** Yanfei Shen, Juqin Shao

**Affiliations:** Intensive Care Unit, Zhejiang Hospital, Gudun Road 1229#, Hangzhou, 310013 Zhejiang People’s Republic of China

To the Editor,

In a recent study, Dr. Polok et al. [1] investigated the potential efficacy of noninvasive ventilation (NIV) in COVID-19 patients aged ≥ 70 years. They reported that primary NIV failure occurred in 74.7% (470/629) of these patients, and compared to primary invasive mechanical ventilation (IMV), primary NIV was significantly associated with increased mortality. This study adds great information to the current knowledge. However, several points should be noted when interpreting these findings.

NIV has been wildly used in COVID-19 patients. However, the efficacy varied greatly within different areas and COVID-19 periods. In the current study, the NIV failure rate is as high as 74.7%. Noteworthy, patients included in this study were recruited between March 2020 and April 2021, during which the medical resource was extremely limited (early stage of the COVID-19 break). During that period, the shortage of intensive care unit (ICU) beds required many COVID-19 patients to be treated outside the ICU despite severe gas exchange impairment, and NIV may be used as a surrogate of IMV. In this case, NIV use may delay necessary intubation and lung protective ventilation in patients with severe hypoxemia, and increase the risk of aspiration pneumonia [2].

Coppadoro et al. [3] compared the NIV efficacy between patients receiving full treatment support and those with limited medical care resources. A total of 306 patients were included, and the NIV failure rate in patients with limited medical care resources was similar to the current study (72% vs. 74%), and significantly higher than those with full treatment support (93/130 (72%) vs. 54/176 (31%), *p* < 0.001). In addition, another multicenter, randomized trial [4] performed at the late stage of COVID-19 (February 2021–November 2021, low risk of medical resource shortage) reported that 47% (75/159) of patients in the NIV group received endotracheal intubation, and the 28-day mortality was only 27%. Therefore, it is reasonable to infer that the high NIV failure rate in the current study may be caused by potential medical resource shortages and inappropriate application of NIV. Whether NIV can improve prognosis (timing and protocol) without medical resource shortage in COVID-19 needs further investigation [5].

Second, we also have some different opinions on the comparison between primary IMV and NIV. In the sensitivity analysis, we note that compared to primary IMV, NIV increased the mortality rate in patients with life-sustaining limitations (withheld or withdrawn). Withheld or withdrawn from life-sustaining usually represents a severe clinical condition (e.g., severe hypoxemia), in which NIV may be inappropriate. However, in patients without life-sustaining limitations, NIV and IMV showed similar mortality rates. Therefore, whether primary NIV was associated with worse outcomes than primary IMV remains uncertain.

## Data Availability

Not applicable.
